# Supplementary material for: Epigenetic Regulation of Estrogen Receptor Genes’ Expressions in Adipose Tissue in the Course of Obesity
Source: Int J Mol Sci. 2022 May 26;23(11):5989. doi: 10.3390/ijms23115989 (PMC9181405; doi:10.3390/ijms23115989)
Supplement: Supplementary file 1 [file ijms-23-05989-s001.zip › ijms-1732738-supplementary.pdf]

Supplementary Data

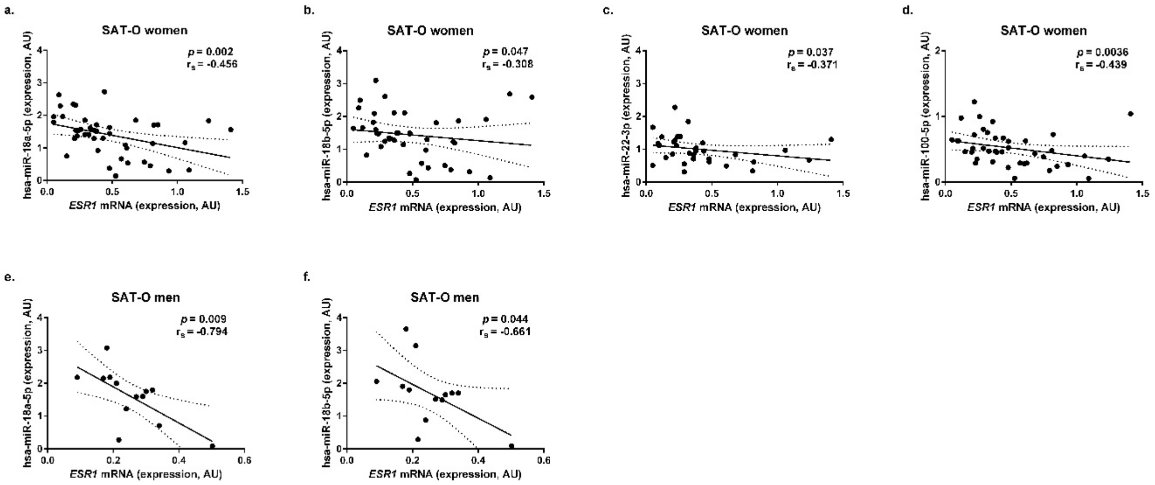

Figure S1. Correlations of *ESR1* mRNA concentrations with hsa-miR-18a-5p (**a**), hsa-miR-18a-5p (**b**), hsa-miR-22-3p (**c**), and hsa-miR-100-5p (**d**) levels in the subcutaneous adipose tissue (SAT) of female obese study participants and with hsa-miR-18a-5p (**e**), and hsa-miR-18b-5p (f) in the SAT of male obese study participants.

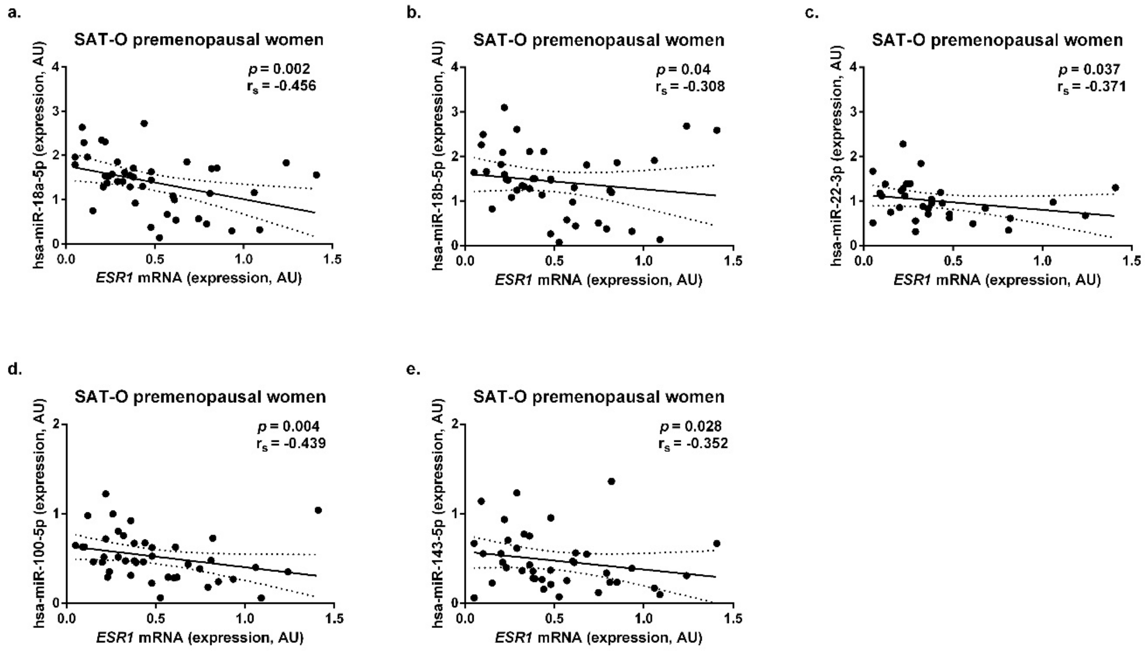

Figure S2. Correlations between *ESR1* mRNA concentrations and hsa-miR-18a-5p (**a**), hsa-miR-18b-5p (**b**), hsa-miR-22-3p (**c**), hsa-miR-100-5p (**d**), and hsa-miR-143-5p (**e**) levels in the subcutaneous adipose tissue (SAT) of premenopausal female obese (O) study participants.

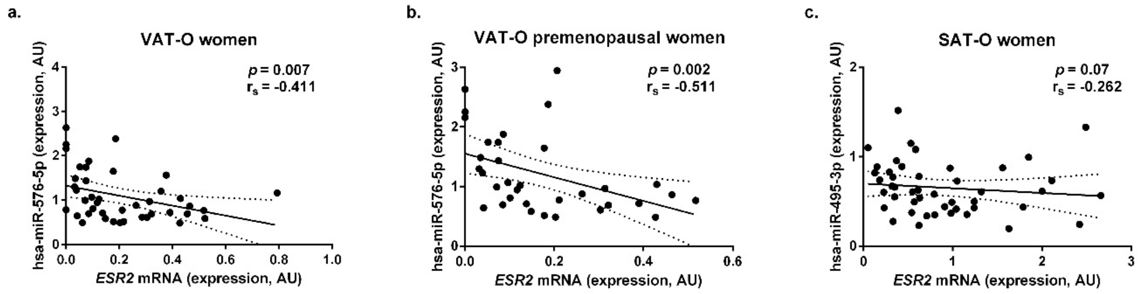

Figure S3. Correlations of *ESR2* mRNA concentrations with hsa-miR-576-5p levels in the visceral adipose tissue (VAT) of obese (O) female study participants (**a**), hsa-miR-576-5p levels in VAT of obese (O) premenopausal female study participants (**b**), and hsa-miR-495-3p levels in subcutaneous adipose tissue (SAT) of obese (O) female study participants (**c**).

Table S1. Primers used for the mRNA, miRNA and methylation analysis.

Gene		Primers
ESR1	**F**	5′ CCGGCTCCGCAAATTGCT 3′
	**R**	5′ AGCGGGTTGGCCAAAGGTT 3′
ESR2	**F**	5′ GACAGCGCAGAAGTGAGCATC 3′
	**R**	5′ AAGCCGTGATGTAAAGGGTG 3′
ACTB	**F**	5′ CAGCCTGGATAGCAACGTAC 3′
	**R**	5′ TTCTACAATGAGCTGCGTGTG 3′
**miRNA**		
hsa-miR-18a-5p	**F**	5′ GTAAGGTGCATCTAGTGCAG 3′
	**R**	5′ GGTCCAGTTTTTTTTTTTTTTTCTATC 3′
hsa-miR-18b-5p	**F**	5′ GTAAGGTGCATCTAGTGCAG 3′
	**R**	5′ GGTCCAGTTTTTTTTTTTTTTTCTAAC 3′
hsa-miR-20b-5p	**F**	5′ GCAAAGTGCTCATAGTGCAG 3′
	**R**	5′ GGTCCAGTTTTTTTTTTTTTTTCTAC 3′
hsa-miR-22-3p	**F**	5′ GAAGCTGCCAGTTGAAGA 3′
	**R**	5′ GGTCCAGTTTTTTTTTTTTTTTACAGT 3′
hsa-miR-100-5p	**F**	5′ CAGAACCCGTAGATCCGA 3′
	**R**	5′ GTCCAGTTTTTTTTTTTTTTTCACAAG 3′
hsa-miR-142-3p	**F**	5′ CGCAGTGTAGTGTTTCCT 3′
	**R**	5′ GGTCCAGTTTTTTTTTTTTTTTCCA 3′
hsa-miR-143-5p	**F**	5′ TGCAGTGCTGCATCTCT 3′
	**R**	5′ GGTCCAGTTTTTTTTTTTTTTTACCA 3′
hsa-miR-146b-3p	**F**	5′ GCCCTGTGGACTCAGT 3′
	**R**	5′ GTCCAGTTTTTTTTTTTTTTTACCAGA 3′
hsa-miR-335-3p	**F**	5′ CGCAGTTTTCATTATTGCTCCT 3′
	**R**	5′ GGTCCAGTTTTTTTTTTTTTTTGGT 3′
hsa-miR-495-3p	**F**	5′ CGCAGAAACAAACATGGTG 3′
	**R**	5′ GTCCAGTTTTTTTTTTTTTTTAAGAAGTG 3′
hsa-miR-576-5p	**F**	5′ GCAGATTCTAATTTCTCCACGTC 3′
	**R**	5′ CAGGTCCAGTTTTTTTTTTTTTTTAAAG 3′
**Gene**		
ESR1 1st fragment (-34 bp to +255 bp)	**F**	5′ TCCTCCAGCACCTTTGTAATG 3′
	**R**	5′ GTGTGGAGGGTCATGGTCAT 3′
ESR1 2nd fragment (+255 to +615 bp)	**F**	5′ ATGACCATGACCCTCCACAC 3′
	**R**	5′ TCCAGGTAGTAGGGCACCTG 3′
ESR2 P0K 1st fragment (-56 044 to -55 433 bp)	**F**	5′ CTCCTGTGGTCCAACATTCC 3′
	**R**	5′ TCCACGTTTTCCTTGTCCTC 3′
ESR2 P0K 2nd fragment (-56 044 to -55 433 bp)	**F**	5′ GACTGGCTGAAGGGGATGAT 3′
	**R**	5′GCCCTCACACTTCTGCAC 3′
ESR2 P0N 1st fragment (-11 532 to -10 258 bp)	**F**	5′ TTCTCAGGCCCTACTCCAGA 3′
	**R**	5′ CAGCAGCTGGAGAAACTGAA 3′
ESR2 P0N 2nd fragment (-11 194 to -10 891 bp)	**F**	5′ AACTGCCTCTTGAAACTTGC 3′
	**R**	5′ CAGGATTTCAAACAAAATGAGACA 3′

bp – base pairs, F – forward, P0K – distal promoter, P0N – central promoter, R – reverse

Table S2. The mean percentage of the methylated CG pairs in the *ESR1* and *ESR2* regulatory regions

Analysed gene fragment	Length (bp)	CpG (N)	VAT-O	SAT-O	VAT-O MS	SAT-O MS	VAT-O NOMS	SAT-O NOMS	VAT-N	SAT-N
ESR1 1st fragment	289	28	63.2%	51.6%	60.9%	50.1%	65.5%	53.2%	62.4%	54.7%
ESR1 2nd fragment	360	34	58.8%	53.5%	59.7%	54.6%	57.9%	52.4%	65.1%	59.9%
ESR2 P0K 1st fragment	275	21	3.5%	1.8%	2.6 %	1.9%	4.4%	1.7 %	2.2%	1.8%
ESR2 P0K 2nd fragment	322	28	1.7%	1.7%	1.8%	1.9%	1.6%	1.5%	1.6%	1.6%
ESR2 P0N 1st fragment	277	16	15.3%	14.0%	14.5%	16.0%	16.1%	12.1%	15.2%	17.4%
ESR2 P0N 2nd fragment	303	25	11.6%	12.2%	10.6%	12.0%	12.1%	12.4%	11.5%	11.4%

bp – base pairs, MS – metabolic syndrome (patients with metabolic complications of obesity), NOMS – no metabolic syndrome components (metabolically healthy), N – number, P0K – distal promoter, P0N – central promoter,
